# Gamma Oscillations of Spiking Neural Populations Enhance Signal Discrimination

**DOI:** 10.1371/journal.pcbi.0030236

**Published:** 2007-11-30

**Authors:** Naoki Masuda, Brent Doiron

**Affiliations:** 1 Graduate School of Information Science and Technology, the University of Tokyo, Bunkyo, Tokyo, Japan; 2 Amari Research Unit, RIKEN Brain Science Institute, Wako, Saitama, Japan; 3 Center for Neural Science, New York University, New York, New York, United States of America; University College London, United Kingdom

## Abstract

Selective attention is an important filter for complex environments where distractions compete with signals. Attention increases both the gamma-band power of cortical local field potentials and the spike-field coherence within the receptive field of an attended object. However, the mechanisms by which gamma-band activity enhances, if at all, the encoding of input signals are not well understood. We propose that gamma oscillations induce binomial-like spike-count statistics across noisy neural populations. Using simplified models of spiking neurons, we show how the discrimination of static signals based on the population spike-count response is improved with gamma induced binomial statistics. These results give an important mechanistic link between the neural correlates of attention and the discrimination tasks where attention is known to enhance performance. Further, they show how a rhythmicity of spike responses can enhance coding schemes that are not temporally sensitive.

## Author Summary

Rhythmic brain activity is observed in many neural structures and is an inferred critical component of neural processing. In particular, stimulus induced oscillations in the gamma-frequency band (30–80 Hz) are common in several cortical networks. Many experimental and theoretical studies have established the neural mechanisms by which a population of neurons produce and control gamma-band activity. However, the beneficial role, if any, of gamma activity in neural processing is rarely discussed. It is increasingly apparent that gamma oscillatory power increases with subject attention to a sensory scene. Attention is associated with enhanced performance of discrimination tasks, where relevant stimuli compete with distracters. In this study we explore how gamma-band activity serves to enhance the discrimination of stimuli. We use computational models to show that the gamma rhythmicity in a population of spiking neurons drastically reduces the response variability when a preferred stimulus is present. This drop in response variability enhances stimulus discrimination and increases the overall information throughput in sensory cortex. Our results provide a much-needed link between the dynamics of neural populations and the coding tasks they perform, as well as give insight on why—rather than how—attention mediates gamma activity.

## Introduction

Past work with both human and animal subjects has focused on neural correlates of attention. Attention raises the firing rate and the input–output gain of orientation-selective neurons in the visual cortex [1–3], and shifts response curves so that physiologically relevant stimuli fall in the high-gain region [4,5]. Also, when attended stimuli overlap with a recorded receptive field, gamma-band frequency components (30–80 Hz) of local field potentials and single-unit spike responses increase [6–10]. Gamma oscillations in the field potential likely reflect correlated network activity [7,9], as supported by simulations of spiking neurons with inhibitory or recurrent excitatory–inhibitory coupling [11]. Attention is thought to influence cholingergic neuromodulation [12], which presumably affects synchrony of interneuron networks involved in gamma oscillations [11,13,14]. It is well-known that correlated network discharge effectively drives postsynaptic cells [15], making gamma-band activity a signature of efficient signal propagation. This would allow attended objects to increase downstream responses, as compared to nonattended objects. In contrast, we assess the role of gamma oscillations in the signal *coding* of neural populations participating in gamma oscillatory dynamics.

Tasks where attention improves performance typically involve discrimination between different signals, such as visual cues with different colors, shapes, or orientations [1,6–10]. Although there are a large number of studies exploring how gamma rhythms are generated in networks of spiking neurons (for a review, see [11]), the mechanisms by which gamma oscillations modify signal discrimination are elusive in three aspects. First, the relation between gain modulation and gamma oscillations, both of which are attention-dependent, is unclear. Second, the temporal relation between a network gamma rhythm and the time course of a driving signal is often unclear. Third, gamma-induced synchronous firing may be deleterious for coding due to increased variability of population activity [16].

A popular framework for neural coding is that the number of spikes produced by a single neuron or a population of neurons carries information about a driving signal. However, in vivo spike trains often show a spike count Fano factor (ratio of the spike-count variance to the mean spike count) that is close to or even exceeds unity [16–18]. This trial-to-trial variability is deleterious to the code performance and degrades putative spike-count–based signal-discrimination schemes. In certain situations, Fano factors much less than 1 are observed in the visual cortex [19,20], the auditory cortex [21], and the salamander retina [22]. In an extreme case, if a neuron fires with high probability in response to a relevant input signal and rarely fires otherwise [21], then the signal can be estimated from the spike count with small error. In addition, spike-*timing* reliability, for which a neuron robustly emits just a single spike during a steep upstroke of the input and seldom fires elsewhere [23,24], is also supportive of such binary spiking.

In this study we model the essence of a gamma frequency modulation as a simple rhythmic forcing of a population of uncoupled spiking neurons. We show that gamma oscillations endow population spike counts with binomial-like statistics, which improve signal discrimination over a range of stimuli through reduced spike-count variability. In this way, we propose a connection between gamma oscillations and enhanced task performance found in behavioral experiments. Our results are both distinct and complementary to previously described influences of rhythmic network behavior in temporal coding schemes by improving spike precision [25,26] or by providing a clock for a phase-based code [27–30].

## Results

### Gamma-Induced Binomial Statistics

We consider signal discrimination tasks using a population of *N* = 100 uncoupled leaky integrate-and-fire (LIF) neurons (see Methods). The input to each neuron *I*(*t*) is the sum of the input signal *s*, the gamma modulation, and a fast fluctuating noise term:

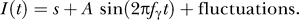



For simplicity, we take the fluctuations to be broadband (e.g., white noise) with intensity *σ* and correlation coefficient *c* between neuron pairs in the population [31]. We assume that the fluctuations are correlated among neurons to comply with experimental evidence [16] and to make our discrimination task somewhat difficult, thereby allowing gamma activity to shape the results. We remark that we simply force each neuron with a sinusoidal current with amplitude *A* and frequency *f_γ_ =* 40 Hz , rather than explicitly model the gamma oscillation as emergent from neural networks (see [11]).

We examine the statistics of the population spike count 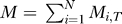 , where *M_i,T_* is the number of times neuron *i* spikes in a window of length *T*. In an observation window, each neuron can fire an arbitrary number of times with a maximum of *T/*τ*_r_*, where τ*_r_* is the absolute refractory period. If the firing rate approaches this upper limit, presumably by a large *I*(*t*), all neurons fire regularly with period close to τ*_r_*, and *M* has low variance. However, 1/τ*_r_* is typically hundreds of Hz, making such a saturation unreasonable for prolonged times. It is well known that the relative refractory periods enable low spike-count variability at moderate firing rates [20,22]. We explore an alternative possibility that gamma oscillations generate regular spiking at firing rates far below 1/τ*_r_* when the observation window *T* is sufficiently large. In what follows, for simplicity we take *T* = 1/*f_γ_*.

**Figure 1:**
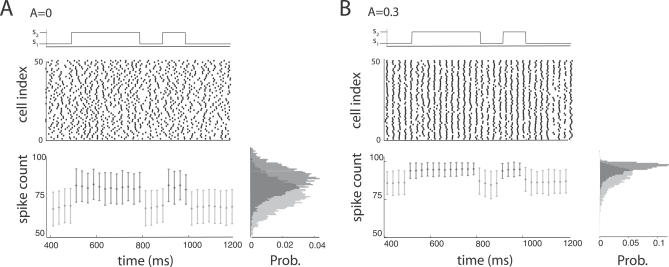
Population Activity of LIF Neurons with Switching Input Signals (A) Top: input signal *s* switching between two levels, *s*_1_ = 0.99 and *s*_2_ = 1.03. Middle: corresponding spike raster plots for 50 neurons. Bottom: mean spike count *μ* and error bars 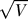 . Here gamma oscillations are absent (*A* = 0). The spike-count variance *V* is computed from 200 realizations of network activity. The *KL_R_* between the spike count responses for *s* = *s*_1_ and *s* = *s*_2_ is 0.343, and the *d*′ is 0.645 (see Methods). (B) Same as (A) but when gamma oscillations are present (*A* = 0.3). The *KL_R_* increases to 0.459 and the *d′* to 0.814, showing enhanced discriminability.

To illustrate how gamma modulation influences population spike-count statistics, we switch the external signal between two static levels *s* = *s*_1_ and *s* = *s*_2_ (Figure 1). In the absence of gamma modulation (*A* = 0), the spike raster (Figure 1A, middle) and the spike count (Figure 1A, bottom) show a subtle but noticeable change in the statistics of *M* as *s* switches between *s*_1_ and *s*_2_. However, with finite observation time, the large trial-to-trial variability (error bars in Figure 1A, bottom) makes discrimination between *s*_1_ and *s*_2_ based on *M* difficult when *s*_1_ and *s*_2_ are close to one another. This difficulty is reflected by a large overlap in the spike-count probability density functions (PDFs) conditioned on *s* = *s*_1_ or *s* = *s*_2_ (Figure 1A, bottom). Further, correlated fluctuations (*c* > 0) bound the population spike-count variability to a nonzero value even for very large populations [16]. In contrast, with moderate *A*, neurons fire at most one spike per cycle because of the rhythmic nature of *I*(*t*) combined with the absolute spike refractory period (Figure 1B, middle). For larger *s* values, the neurons fire once every cycle with high probability, yielding a population spike count with low variability (small error bars in Figure 1B, bottom, for *s* = *s*_2_). The overlap of the two spike count PDFs in this case is actually smaller than that for *A* = 0. Consequently, discriminability between *s*_1_ and *s*_2_ is enhanced by gamma modulation (see Figure 1 caption). The remainder of the paper seeks to quantify this observation. In what follows we let *s*_1_ and *s*_2_ be constant in time; this simplification is reasonable since the observation window *T* is quite short compared to typical time scales of natural stimuli.

**Figure 2:**
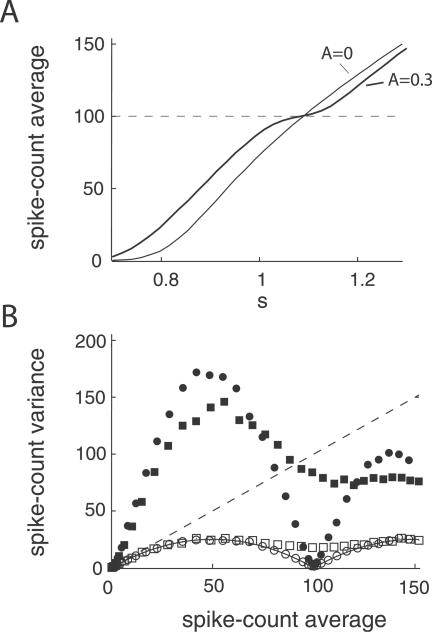
Gamma Oscillations Produce Binomial Spike Statistics for LIF Neurons (A) Mean spike count plotted against static input signals s without (*A* = 0, thin line) and with (*A* = 0.3, thick line) gamma modulation. Note the knee in the curve for mean spike counts of approximately *μ* = 100 (= *N*, dashed line) when gamma modulation is present. (B) Relation between the mean spike count and the spike-count variance for the simulated network without (squares) and with (circles) gamma modulation. Open and closed symbols correspond to *c* = 0 and *c* = 0.12, respectively. The dashed line corresponds to the mean-variance relation for a Poisson distributed spike count, while the solid line for a binomial distributed spike count. For a fixed *s*, we computed the spike-count statistics from 3,000 gamma cycles.

We first examine the relation between the mean spike count *μ* = 〈*M*〉 and the spike-count variance *V* = 〈*M* ^2^〉 − 〈*M*〉^2^, where 〈·〉 is an average over gamma cycles. Figure 2A shows *μ* plotted against *s* for *A* = 0 (thin line) and *A* = 0.3 (thick line). First, the gamma modulation induces a leftward shift in the *μ*–*s* curve for *s* < 1. Second, a knee in the curve near *μ* = *N* (= 100) emerges when *A* > 0, indicating one-to-one locking of single neuron firing and the gamma cycle. The additive shift and the response saturation at moderate rates are both consistent with single-unit spike responses during attention-sensitive tasks (Figure 5A of [4]). To study how the knee region influences count variability, we plot *V* versus *μ* for *A* = 0 and *A* = 0.3 (Figure 2B). When correlated noise is both present (*c* = 0.12; closed symbols) and absent (*c* = 0; open symbols), *V* is smaller with gamma modulation (circles) than without (squares), *conditional* on *s* chosen so that all the neurons fire once in a window with high probability, *μ* ≈ *N* (i.e., in the knee region of the *μ*–*s* curve). When *c* = 0 and *A* = 0.3 (open circles), the relation is well-fit by that for the binomial distribution (solid line), reminiscent of binary spiking statistics for each cell in the population. When *A* = 0 (open squares), *V* does not approach low values for any *μ*. Nevertheless, Poisson count statistics (*V* = *μ*, dashed line), which are in rough agreement with in vivo evidence [17,18], result in a poor fit for large *μ*, because a large *s* transitions the single-cell spiking from a fluctuation driven to oscillatory regime where the large average current drives rhythmic firing (but see Figure 6). These overall trends are preserved when *c* > 0 (closed symbols) in spite of a larger *V*. Our results with *A* > 0 are in agreement with similar numerical studies [14] where gamma oscillations were replicated with realistic barrages of synchronous inhibitory conductances (Figure 4D in [14]).

### Signal Discrimination by Phenomenological Spiking Models

To explore the link between gamma-induced binomial spiking and signal discrimination, we first study phenomenological models of stochastic population activity. We map the signal *s* to an internal parameter that characterizes the spike-count distributions. In a Poisson model we set the expected number of spikes for a single neuron to be *λ* = *s*. If neurons fire independently, then the population spike count follows a Poisson distribution: *P*(*M* = *k*) = *e* ^–*Nλ*^(*Nλ*)*^k^* / *k*!, which gives *μ* = *V* = *N*λ. The Poisson model represents a scenario in which reduction in spike-count variability of any kind is absent. In a binomial model, each neuron fires at most once in the window and does so with probability *p* (0 ≤ *p* ≤ 1). For each neuron, the *s* to *p* relation is a smoothed piecewise map so that for small *s* the map is near linear and as *s* → 1 the population response saturates (i.e., *p* → 1). If all neurons fire independently, *P*(*M* = *k*) = *_N_C_k_p^k^* (1 − *p*)*^N^* ^−*k*^, where *_N_C_k_* is a binomial coefficient. This gives *μ* = *Np* and *V* = *Np*(1 − *p*).

We mimic the effect of attention in either model with an additional internal modulation *s_A_* that modifies the statistics of *M*. Because attention is thought to modulate spike statistics in several ways, we consider two accepted scenarios. One is an additive scenario in which *s* is mapped to *s* + *s_A_*. This is similar to attention-mediated leftward shifts of input–output curves [4,5] in the visual pathway. The other is a multiplicative scenario in which *s* is mapped to *s*(1 + *s_A_*), modeling experiments where attention multiplicatively controls the gain of orientation tuning curves in primary and middle visual areas [2,3]. These two gain manipulation schemes result in similar effects from our spike-count perspective (see below).

To quantify the discriminability of two signals, we consider the conditioned PDFs *P*(*M*|*s*_1_) and *P*(*M*|*s*_2_). Intuitively, discrimination is easier when the masses of the two PDFs are more separated. To assess discriminability, we compute the Kullback-Leibler (KL) distance [32,33] between *P*(*M*|*s*_1_) and *P*(*M*|*s*_2_) (see Methods). In short, the KL distance, which we denote by *KL_R_* (*R* for resistor average, see Methods), offers a method for measuring the distance between two PDFs. For Gaussian PDFs, the KL distance is equivalent to the so-called *d′* discriminability [32], which is often used in psychophysical studies [34]. However, *P*(*M*|*s*_1_) and *P*(*M*|*s*_2_) are generally non-Gaussian, as is the case for binomial spike statistics, and the KL measures are more appropriate. We label *KL_R_* with a subscript P or B for statistics using the Poisson or binomial model, respectively.

**Figure 3:**
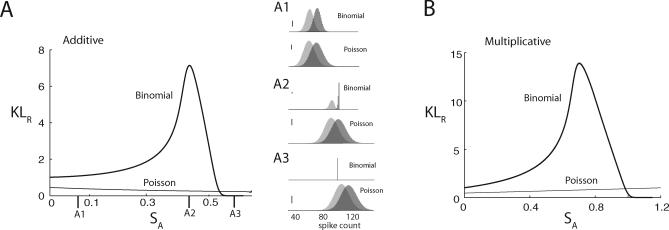
Signal Discrimination by Phenomenological Statistical Models of Spike Activity (A) Comparison of the KL distance between the Poisson and binomial spike-count models where attention is treated as an additive modulation of input so that *s_i_* → *s_i_* + *s_A_* (*i* = 1, 2). Specifically, for two fixed-input signals *s*_1_ and *s*_2_, we define the Poisson model for single-neuron spike counts with parameter *λ_i_* = *s_i_* and the binomial model with parameter 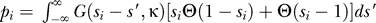 , where *G*(*x*,*κ*) is a Gaussian kernal with mean *x* and variance *κ* = 0.0001 (we smooth the mapping to remove discontinuites in *KL_R_* as *p* → 1). It is straightforward to show that 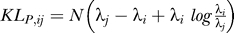 and 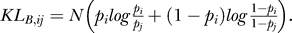 We plot the symmetrized KL distance 
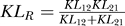
 (see Methods). For the purposes of illustration, we fix *s*_1_ = 0.1 (light grey) and *s*_2_ = 0.2 (dark grey) and plot the conditional PDFs *P*(*M*|*s_i_*) for both the Poisson and binomial models for *s_A_* = 0.075 (inset A1), *s_A_* = 0.4 (inset A2), and *s_A_* = 0.6 (inset A3). The vertical scale bars in the insets represent a probability of 0.01. (B) Comparison of the KL distance when attention is treated as a multiplicative modulation of input so that *s_i_* → *s_i_* (1 + *s_A_*). *KL_P,ij_* and *KL_B,ij_* are the same as above yet with the new *λ_i_*, *λ_j_*, *p_i_*, and *p_j_* inserted.

Motivated by the gamma-induced additive shift in the network simulations shown in Figure 2A, we first focus on the additive model. We vary *s_A_* with *s*_1_ and *s*_2_ fixed, assuming without a loss of generality that *s*_1_ ≤ *s*_2_. For small *s_A_*, we have *μ_B_* ≈ *V_B_*, and thus the binomial and Poisson models are statistically similar, yielding *KL_B,R_* ≈ *KL_P,R_* (Figure 3A). Indeed, for *s_A_* fixed at a small value, the conditional PDF for the Poisson model and those for the binomial model are nearly identical, both for *s*_1_ and *s*_2_ (Figure 3A1). As *s_A_* increases, *KL_B,R_* rises significantly, whereas *KL_P,R_* drops slightly. To understand this, we note that, in the binomial model, when *s*_2_ but not *s*_1_ saturates the population response (i.e., *p*_2_ → 1 and *p*_1_ < 1), the variance of *P_B_*(*M*|*s*_2_) drops significantly to reduce the overlap between *P_B_*(*M*|*s*_1_) and *P_B_*(*M*|*s*_2_) (Figure 3A2). Consequently, signal discrimination becomes easier. In the Poisson model, the population spike-count variability increases with *s_A_*, yielding an increased overlap between *P_P_*(*M*|*s*_1_) and *P_P_*(*M*|*s*_2_), which drops *KL_P,R_*. However, when *s_A_* is even larger, binomial population responses are saturated for both *s*_1_ and *s*_2_ (*p*_1_, *p*_2_ → 1), giving *P_B_*(*M*|*s*_1_) = *P_B_*(*M*|*s*_2_) ≈ δ*_M,N_* , and hence *KL_B,R_* ≈ 0, whereas *KL_P_*_,*R*_ > 0 (Figure 3A3).

In total, as *s_A_* is varied, *KL_B,R_* is non-monotonic, whereas *KL_P_*_,*R*_ monotonically decreases over the same range of *s_A_*. Similar results are obtained for the multiplicative model except that *KL_P,R_* increases slightly with *s_A_* (Figure 3B). In Methods, we generalize these results by showing *KL_B_*_,*R*_ ≥ *KL_P_*_,*R*_ for any *s*_1_ and *s*_2_ pair unless both *s*_1_ and *s*_2_ saturate the binomial model response. Overall, binomial spike-count statistics can enhance signal discrimination as compared to Poisson statistics, particularly when one input signal saturates or nearly saturates the population response while the other signal is below saturation.

### Signal Discrimination Improved by Gamma Oscillations

**Figure 4:**
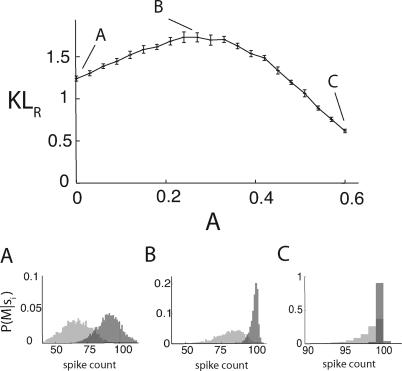
Gamma Enhanced Signal Discriminability KL distance for the population of LIF neurons for (*s*_1_, *s*_2_) = (0.98, 1.06) as the amplitude of the gamma modulation is increased. A distinct non-monotonic trend is apparent. We show the conditional PDFs *P*(*M*|*s_i_*) used to compute *KL_R_* for *A* = 0 (inset A), *A* = 0.27 (inset B), and *A* = 0.6 (inset C). As in Figure 3, light grey corresponds to *s*_1_ while dark grey to *s*_2_. We set *c* = 0.12, and for each value of *A*, we computed the spike-count statistics from 3,000 gamma cycles.

We next link gamma induced binomial-like spike-count statistics of a population of LIF neurons with the discrimination results obtained with the phenomenological models. In the spiking neuron population, we fix *s*_1_ and *s*_2_, as was done in Figure 3, and numerically estimate *P*(*M*|*s*_1_), *P*(*M*|*s*_2_), and the KL distance for a fixed *A*. Interestingly, *KL_R_* is nonmonotonic as *A* ranges from 0 to 0.6 (Figure 4). Specifically, when *A* = 0, *P*(*M*|*s*_1_) and *P*(*M*|*s*_2_) are roughly Gaussian (Figure 4A), and *KL_R_* is about 1.2. As *A* increases, the spike-count statistics become increasingly better described by a binomial random variable (see Figure 2), and *P*(*M*|*s*_2_) shows a reduced variance. This leads to an overall increase in *KL_R_* (Figure 4B). As *A* increases further, the population response is dominated by the gamma oscillation and is saturated at *M* ≈ *N* for both *s*_1_ and *s*_2_ (Figure 4C), ultimately dropping *KL_R_* significantly. This confirms the original hypothesis (Figure 1) that gamma oscillations can enhance signal discrimination of a population of spiking neurons.

A comparison between the non-monotonic trend of *KL_R_* shown in Figure 3 and that shown in Figure 4A should be done with care. In the phenomenological binomial model, the spike statistics were modulated by the attention variable *s_A_*, yet were, by design, binomial for all *s_A_*. In the network simulations, the spike-count statistics become better and better described by a binomial random variable as *A* increases. Although it is tempting to associate *A* with *s_A_*, *A* both shifts the population response statistics from Poisson-like to binomial-like, and at the same time modulates the spike-count statistics, similar to the variables *p* or λ in the phenomenological models. This is a minor point, since for moderate *s*_1_ and *s*_2_, the binomial statistics for small *s_A_* are well-approximated by a Poisson spike count (Figure 3), similar to the case of small *A* in the network simulations. Thus, the basic mechanism of the non-monotonic trend in Figures 3 and 4 is qualitatively the same.

**Figure 5:**
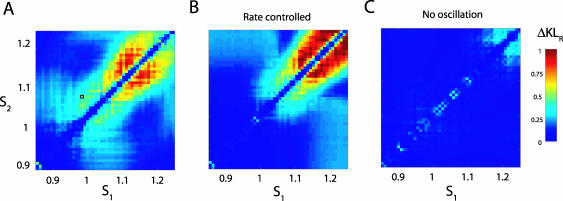
Gamma Enhanced Signal Discriminability in the Population of LIF Neurons for a Range of Input Signal Pairs (A) The relative *KL_R_* change, Δ*KL_R_*, compares the discriminability of the population spike count in the case where *A* maximizes *KL_R_* to that for *A* = 0 (see text). The black square located at (*s*_1_, *s*_2_) = (0.98,1.06) corresponds to the data shown in Figure 4. (B) Same as (A) but that, for each signal pair, the population firing rate is kept roughly constant for different values of *A* by adding an appropriate constant hyperpolarizing current to the membrane equations. (C) Enhancement of signal discrimination through addition of a constant depolarizing current rather than gamma modulation.

To show the robustness of the increases in *KL_R_* with respect to the choice of signals, we vary *s*_1_ and *s*_2_ to cover both subthreshold (*s*_1_, *s*_2_ < 1) and suprathreshold (*s*_1_, *s*_2_ > 1) regimes. The input signal is confined to 0.85 ≤ *s*_1_, *s*_2_ ≤ 1.25, which yields moderate firing rates (8 Hz for *s* = 0.85 and 56 Hz for *s* = 1.25 without gamma modulation). For each signal pair, we determine the value of *A* maximizing *KL_R_*, which we label 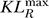 . In Figure 5A, we plot the relative increase in discriminability 
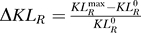
 , where 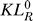 is the value of *KL_R_* in the absence of the gamma. Δ*KL_R_* is large (more than 0.3 as shown in Figure 4) over a wide range, indicating that gamma-enhanced signal discrimination is a general result. The improvement is best manifested when signals are somewhat suprathreshold (1.05 ≤ *s*_1_, *s*_2_ ≤ 1.2) for which the low spike-count variability is induced by gamma oscillations. The improvement is also restricted to near the s_1_-s_2_ diagonal; far off the diagonal, signal discrimination is easy and does not require gamma oscillations.

In Figures 2A and 4, firing rates increased with *A* when *s* is not too large. Indeed, attention often increases firing rates [2–5]. However, in some cases attention raises the gamma-band power without increasing firing rates [7,9]. To show that the improved signal discriminability does not merely result from increased firing rates, we added a negative current bias to the neurons in addition to the gamma modulation so that the firing rates remain constant regardless of *A*. As shown in Figure 5B, Δ*KL_R_* can still be significant, although the range of signal pairs where this is apparent is reduced.

Without gamma oscillations, large static inputs place neurons in the suprathreshold regime, where the net bias drives firing. In this regime, firing is rather regular, and spike-count variability can be low (squares in Figures 2B). To examine the possibility of improved signal discrimination by excess static inputs, we set the gamma frequency *f_γ_* = 0 and shift the phase of the sinusoid by π/2 so that *A* corresponds to an additional bias current. To prevent very large firing rates, we assume 0 ≤ *A* ≤ 0.7. With the largest bias *A* = 0.7, the neurons fire at 81 Hz for *s* = 0.85 and 108 Hz for *s* = 1.25. As shown in Figure 5C, Δ*KL_R_* induced by a constant bias is far less impressive than that by gamma modulation (Figure 5A). Much larger firing rates would considerably increase Δ*KL_R_*, in which case the absolute refractory period of the neurons imposes periodic firing and reduction in spike-count variability, yet prolonged spiking activity at these high rates are not observed in cortical responses. This contrasts to the case with gamma modulation for which neurons fire at most *f_γ_* = 40 Hz. Overall, we conclude that gamma oscillations are an effective means of improving signal discrimination of population responses.

**Figure 6:**
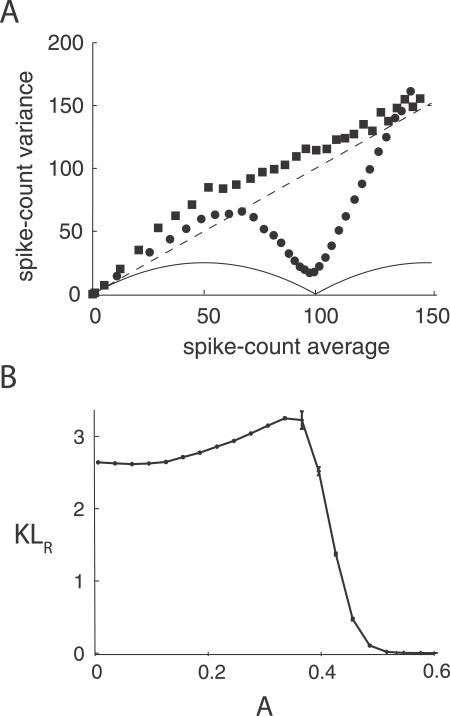
Gamma Enhanced Signal Discriminability in the Population of LIF Neurons with Poisson-Like Spike-Count Statistics (A) Relation between the mean spike count and the spike-count variance without (squares; *A* = 0) and with (circles; *A* = 0.3) gamma modulation. The dashed line corresponds to the relation for a Poisson distributed spike count, while the solid line for a binomial distributed spike count. (B) KL distance for (*s*_1_, *s*_2_) = (0.98,1.06) for a range of the amplitude of the gamma modulation. For each *s* and *A*, we computed the spike-count statistics from 3,000 gamma cycles.

The population of LIF neurons used in Figures 4 and 5 produce small spike-count variances for large firing rates. This relation between the spike-count variance and the spike-count average in the absence of gamma oscillations (closed squares in Figure 2B) deviates from the Poisson relation (dashed line in Figure 2B) observed in many experiments [17,18]. To show the generality of our results, we mimicked more Poisson-like population spike-count statistics by making the input noise temporally colored, scaling the input noise intensity as the square root of the input signal, and increasing the input correlation linearly in the input signal. The first modification assumes a synaptic filter, while the last two model a presynaptic population's tendency to have both the spike-count variance and correlation grow with the mean spike count, as suggested by [17] and [31], respectively. With these modifications, the population spike-count variance in the absence of gamma oscillations is roughly equal to the spike-count average for a wide range of the firing rate (squares in Figure 6A). Also in this situation, the spike-count variance sharply drops near *M* = *N* with gamma modulation (circles in Figure 6A). Accordingly, and similar to our earlier model (Figure 4), signal discrimination improves for intermediate gamma amplitudes, as shown in Figure 6B. These final results show that gamma-enhanced signal discrimination is robust to significant changes in population response statistics.

## Discussion

We have shown that gamma modulation of a population of noisy spiking neurons imparts binomial-like spike-count statistics. When neurons are driven to fire at rates near gamma frequency, they phase lock with the gamma oscillation. This produces a saturation of the firing rate, reduction of spike-count variability, and importantly enhanced signal discriminability. Simple phenomenological statistical models (Figure 3) show this to be a straightforward consequence of binomial count statistics. The overall effect is robust in simulations of a population of spiking neurons (Figures 4 *–* 6).

Although we used a simple sine wave forcing as a caricature of gamma activity, experimentally measured gamma oscillations are not harmonic, and are typically broadband (30–60 Hz). Indeed, the spectral properties of the spike-train responses from our model have artificially large spike-train power at 40Hz, and a spike–spike coherence [8] value of approximately 0.5 at 40 Hz, much larger than is typically seen in vivo [8,9]. If we instead used a gamma forcing defined over a range of frequencies, then the large population rhythmicity and coherence at 40 Hz would be spread over a wider spectrum, and no single frequency would be overly dominant. We expect that such a broadband gamma modulation would not deteriorate signal discrimination because it can still elicit approximately one spike per gamma cycle, provided that the gamma band is not too broad and other sources of noise are weak, as shown in the more realistic gamma network model presented in [14]. We stress that our spiking network is only a qualitative description of gamma oscillatory neural dynamics, and not a quantitative description of cortical or hippocampal networks. The robustness of our results to changes in input *s* (Figure 5), changes in input statistics (Figure 6), as well as our simplified phenomenological description (Figure 3), suggests that our result may be operable in many different networks with varying response statistics.

For our theory to be operative, gamma-band activity must be exclusive to a specific subpopulation of neurons involved in a discrimination task. Our theory does not explain how such a selective gamma activity is produced. However, in support of selective modulation of gamma activity, a recent study in area LIP in the parietal cortex gives attention-related feedback projections in the gamma range to MT, which in turn feeds back to V1 [35]. A topographic overlap of feedback architecture and feedforward receptive field would therefore permit a feedback gated selective gamma response.

We dealt with population spike counts whose time resolution was quite low (*T* = 1/*f_γ_* = 25 ms) compared to millisecond precision on which many spike-based temporal coding schemes are based. On shorter time scales (1–5 ms), oscillatory input, for example, enhances spike-time precision by cellular resonances [24] and resets the membrane potential for improved signal discriminability [26]. Oscillatory inputs also set a rhythm for defining spike phases, which are potentially useful for coding [27–29]. These results typically assume that the downstream decoding cells are sensitive to the precise timing of input spikes. Our results are quite complementary because oscillatory activity of the same presynaptic neural populations enhances coding where decoding neurons integrate incoming spikes on much longer timescales (20–30 ms). With different kinetics of downstream neurons and synapses, both coding schemes may act in parallel.

Attention can raise firing rates [2,3], contrast gain [4,5], and gamma-band activity in both spike trains and field potentials [6,7]. In our spiking network, regardless of whether gamma activity increases firing rates or not, signal discrimination is facilitated by gamma modulation that we interpret to be generated by attention. Also in our phenomenological models, when attention is either additive or multiplicative modulation of response properties, a shift from Poisson to binomial spike statistics improves signal discrimination. This is consistent with the recent observations that attention decreases spike-count variability [36], as well as enhances the signal-to-noise ratio [37]. Thus we provide an important link between the dynamical effects of gamma oscillations and coding performance of neural populations that are attention-sensitive.

## Methods

### Network model.

The dynamics of the *i*-th neuron in the population (1 ≤ *i* ≤ *N*) is described by

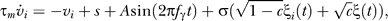
where *v_i_* is the membrane potential of the *i*-th neuron in the population, and τ*_m_* = 10 ms is the membrane time constant. The correlation coefficient between the total background inputs given to two cells is denoted by *c* [33]. We set *c* = 0.12 unless otherwise stated, so that the neurons have a background correlation similar to in vivo recordings in the absence of gamma modulation [16]. The neuron fires when *v_i_* = 1 is reached from below, and then *v_i_* is instantaneously reset to the resting potential equal to 0. The absolute refractory period τ*_r_* is set 2 ms. For Figures 1–5, we let the fluctuation terms *ξ_i_* and *ξ* be uncorrelated white noise inputs with zero mean 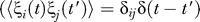 and 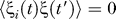 ). The total intensity of these inputs is *σ* = 0.35. In Figure 6, we replace the white noise terms *ξ_i_* and *ξ* with an Ornstein-Uhlenbeck process (low-pass filtered white noise) with a decay time constant of 5 ms. Then we regard that the minimum input signal *s* is equal to 0.85 and scale the input noise intensity and the input correlation as 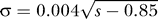 and 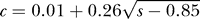 , respectively. We employ a Euler-Maruyama [38] numerical integration scheme (*dt* = 0.02 ms) to solve the population dynamics.

### Population discriminability.

Given two conditional spike-count densities *P*(*M*|*s*_1_) and *P*(*M*|*s*_2_), we compute the Kullback-Leibler divergence [32,33] as

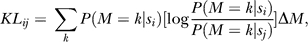
where (*i*,*j*) = (1,2), (2,1). Here *k* ranges over possible spike counts, and Δ*M* = 1 because the spike count is integer-valued. The KL divergence is generally asymmetric, i.e., *KL*_12_ ≠ *KL*_21_. To correct for this, we use the KL distance, or so-called resistor average [32], defined by

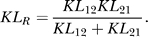



The KL distance approximates the optimal discrimination error better than the simple average (*KL*_12_ + *KL*_21_)/2 does [32]. A direct computation of *KL_ij_* diverges if *P*(*M = k*|*s_i_*) > 0 and *P*(*M = k*|*s_j_*) = 0 for some *k* due to numerical sampling. To accurately estimate the conditional PDFs and the KL distance, we employ the K–T estimate method [32], where 0.5 is added to all the bins in the count histogram before normalization to obtain the PDFs.

### KL measures are larger for the binomial distribution than for the Poisson distribution.

We prove that the KL divergence and the KL distance for the binomial distribution are larger than those for the Poisson distribution when at least one of the stimuli *s*_1_ and *s*_2_ does not saturate the binomial model response.

For the Poisson distributions with parameters *Nλ*_1_ and *Nλ*_2_, we obtain

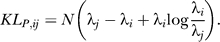
For the binomial distributions with parameters *p*_1_ and *p*_2_ (0 ≤ *p*_1_, *p*_2_ ≤ 1) for a single neuron, we obtain

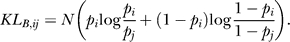
Although we smoothed the *s*-*p* relationship of the binomial model to produce Figure 3, the smoothing function had a very small variance. Therefore, we neglect smoothing so that *p* = *s* for 0 ≤ *s* ≤ 1 and *p* = 1 for *s* > 1. We equate *λ*_1_ = *p*_1_ and *λ*_2_ = *p*_2_ so that the Poisson and binomial distributions produce the same average firing rates. Using Jensen's inequality, we derive

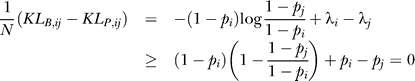
where the equality holds if *p*_1_ = *p*_2_, or equivalently, *λ*_1_ = *λ*_2_. Finally, we obtain

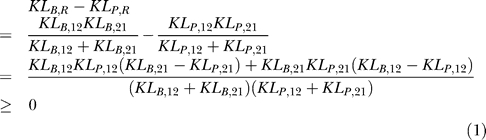
These relations hold when 0 < *p*_1_, *p*_2_ < 1. If *p*_1_ or *p*_2_, but not both, is equal to 0 or 1, *KL*_*B*,12_ or *KL*_*B*,21_ goes to infinity. Even in this case, *KL_B,ij_* ≥ *KL_P,ij_* and *KL_B,R_* ≥ *KL_P,R_* hold. If *p*_1_ = *p*_2_ = 1, the two binomial distributions become delta functions so that *KL_B,ij_* = *KL_B,R_* = 0.

## Abbreviations

LIF: leaky integrate-and-fire
PDF: probability density functions
